# Supplementary material for: Entanglement of photons in their dual wave-particle nature
Source: Nat Commun. 2017 Oct 13;8:915. doi: 10.1038/s41467-017-01058-6 (PMC5688178; doi:10.1038/s41467-017-01058-6)
Supplement: Supplementary file 1 — Supplementary Information [file 41467_2017_1058_MOESM1_ESM.pdf]

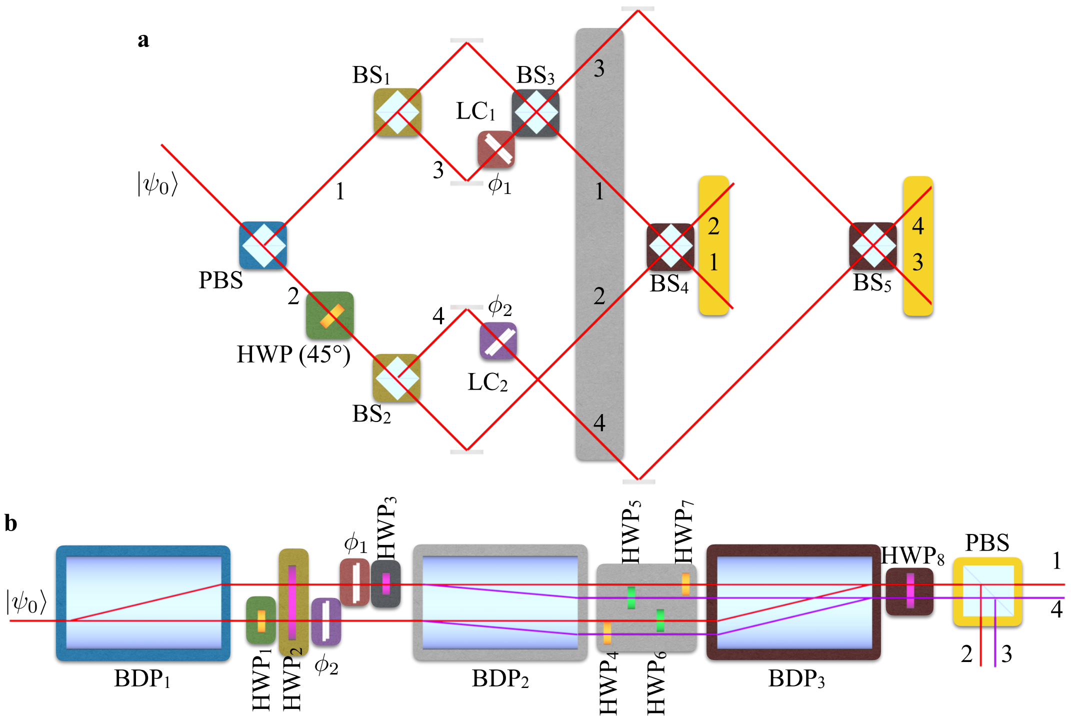

Supplementary Figure 1: **a**, Conceptual scheme of the wave-particle toolbox. **b**, Layout of the experimental implementation of the wave-particle toolbox. Angles of the optical axis orientations for the half-wave plates are: $\text{HWP}_1(45°)$, $\text{HWP}_2(22.5°)$, $\text{HWP}_3(22.5°)$, $\text{HWP}_4(45°)$, $\text{HWP}_5(0°)$, $\text{HWP}_6(0°)$, $\text{HWP}_7(45°)$. Colours below the elements of the two panels identify the analogies between the two schemes.

SUPPLEMENTARY NOTE 1: DESCRIPTION OF THE WAVE-PARTICLE TOOLBOX

We now discuss the implementation of the wave-particle toolbox reported in Fig. 2c-d of the main text. The conceptual scheme (Fig. 1 in the main text) is reported in Supplementary Fig. 1a, while the actual layout is shown in Supplementary Fig. 1b. The implemented toolbox exploits simultaneously the polarization degree of freedom and different spatial modes in an interferometrically stable configuration.

The input state $|\psi_0\rangle$ is separated in two parallel beams according to their polarization state by the first beam-displacing prism (BDP_1), thus implementing the action of the first PBS. Then, HWP_1 with optical axis at $45°$ is placed on the bottom mode of the interferometer. The second half-wave plate (HWP_2) implements simultaneously in the polarization degree of freedom the action of BS_1 and BS_2. After insertion of phases ϕ_1 and ϕ_2 between polarization states through liquid crystals LC_1 and LC_2, the action of BS_3 is reproduced by HWP_3 intercepting only the top mode. Then, beam-displacing prism BDP_2 and the set of half-wave plates HWP_4-HWP_7 is inserted to separate and prepare the four output modes. Finally, the modes are recombined spatially by BDP_3 and in polarization by HWP_8. Depending on the angle of HWP_8, this corresponds to removing ($\beta = 0°$) or inserting ($\beta = 22.5°$) the final beam-splitters BS_4 and BS_5 of the conceptual scheme. The final PBS in the experimental scheme of Supplementary Fig. 1b spatially separates the four output modes 1-4, which are measured by detectors D_1-D_4.

SUPPLEMENTARY NOTE 2: SINGLE-PHOTON WAVE-PARTICLE STATE

In this Section we describe the derivation of the wave-particle superposition state of a single photon which travels along the theoretical scheme, reported in Fig. 1 of the main text (see also Supplementary Fig. 1 above in Supplementary Note 1) and realized by the experimental setup (wave-particle toolbox) of Fig. 2 of the manuscript.

A photon is initially prepared in a polarization state

$$|\psi_0\rangle = \cos\alpha\,|\mathrm{V}\rangle + \sin\alpha\,|\mathrm{H}\rangle\,, \tag{1}$$

with $|\mathrm{V}\rangle$ and $|\mathrm{H}\rangle$ representing the states of vertical and horizontal polarization, respectively. This state is experimentally realized by a half-wave plate (HWP) not shown in Fig. 1 (but evidenced in Fig. 2a in the manuscript). The photon so prepared is sent to a polarizing beam splitter (PBS). Since the vertical polarization of the photon is reflected by the PBS (path 1), while horizontal polarization is transmitted through the PBS (path 2), the photon passes through the upper (wave-like) path of Fig. 1 with a probability amplitude $\cos\alpha$ and it crosses the lower (particle-like) path with a probability amplitude $\sin\alpha$. A HWP ($45°$) is placed after the PBS to obtain equal polarizations between the two spatial modes (paths). Therefore, after the PBS and HWP, the photon state is

$$|\psi_1\rangle = \cos\alpha\,|1\rangle + \sin\alpha\,|2\rangle\,, \tag{2}$$

where $|n\rangle$ ($n = 1, 2, 3, 4$) represents a state of a photon traveling along path n. Then, each path further bifurcates at a balanced beam splitter (BS), BS_1 for path 1 and BS_2 for path 2, transforming $|\psi_1\rangle$ into

$$|\psi_2\rangle = \cos\alpha\left[\frac{1}{\sqrt{2}}(|1\rangle + e^{i\phi_1}|3\rangle)\right] + \sin\alpha\left[\frac{1}{\sqrt{2}}(|2\rangle + e^{i\phi_2}|4\rangle)\right], \tag{3}$$

where ϕ_1 (ϕ_2) is a relative phase introduced by a phase shifter placed in path 3 (4). Notice that paths 3 and 4 are the paths reflected by BS_1 and BS_2, respectively. Paths 1 and 3 are then recombined by BS_3, after which the state $|\psi_2\rangle$ becomes

$$|\psi_{\mathrm{f}}\rangle = \cos\alpha\,|\mathrm{wave}\rangle + \sin\alpha\,|\mathrm{particle}\rangle\,, \tag{4}$$

where

$$|\mathrm{wave}\rangle = e^{i\phi_1/2}(\cos\frac{\phi_1}{2}|1\rangle - i\sin\frac{\phi_1}{2}|3\rangle), \quad |\mathrm{particle}\rangle = \frac{1}{\sqrt{2}}(|2\rangle + e^{i\phi_2}|4\rangle). \tag{5}$$

As mentioned in the main text, this photon state is a coherent superposition of two operational wave and particle states representing, respectively, the ability and inability to produce interference. Photon counting probabilities at detectors D_1, D_3 placed at the end of paths 1, 3 will reveal a wave-like behaviour with their dependence on the phase ϕ_1; on the other hand, photon counting probabilities at detectors D_2, D_4 placed at the end of paths 2, 4 will exhibit a particle-like behaviour independent of the phase ϕ_2. However, these counting probabilities would not allow us to distinguish the wave-particle morphing corresponding to the coherent superposition of equation (4) from that corresponding to the classical incoherent state $\rho_{\mathrm{f}} = \cos^2\alpha\,|\mathrm{wave}\rangle\langle\mathrm{wave}| + \sin^2\alpha\,|\mathrm{particle}\rangle\langle\mathrm{particle}|$ with probabilities $\cos^2\alpha$ and $\sin^2\alpha$, respectively. It is like measuring the state $|\psi_{\mathrm{f}}\rangle$ only along the orthogonal basis $\{|\mathrm{wave}\rangle, |\mathrm{particle}\rangle\}$ corresponding to wave or particle behaviour.

In order to observe a coherent wave-to-particle morphing as a function of α, the wave and particle operational states must interfere at the detection level. This is done by letting paths 1 and 2 synchronize at BS_4, while paths 3 and 4 synchronize at BS_5 in the detection part of the experimental apparatus. The photon state $|\psi_{\mathrm{det}}\rangle$ after these additional beam-splitters results to be

$$|\psi_{\mathrm{det}}\rangle = \cos\alpha|\text{wave-det}\rangle + \sin\alpha|\text{particle-det}\rangle, \tag{6}$$

where

$$\begin{aligned} |\text{wave-det}\rangle &= \frac{e^{i\phi_1/2}}{\sqrt{2}}(\cos\frac{\phi_1}{2}|1\rangle + \cos\frac{\phi_1}{2}|2\rangle - i\sin\frac{\phi_1}{2}|3\rangle - i\sin\frac{\phi_1}{2}|4\rangle), \\ |\text{particle-det}\rangle &= \frac{1}{2}(|1\rangle - |2\rangle + e^{i\phi_2}|3\rangle - e^{i\phi_2}|4\rangle). \end{aligned} \tag{7}$$

Now, as explicitly reported in the main text, the photon counting probabilities at detectors D_n ($n = 1, 2, 3, 4$) reveal the quantum superposition of wave and particle behaviours and the wave-to-particle morphing. The insertion of BS_4 and BS_5 in fact permits one to change the measurement basis into the coherent superposition of wave and particle behaviours. In the manuscript, we notice that the terms $\mathcal{I}_{\mathrm{c}}$, $\mathcal{I}_{\mathrm{s}}$ of the detection probabilities exclusively stem from the interference between the $|\text{wave}\rangle$ and $|\text{particle}\rangle$ components appearing in the generated superposition state $|\psi_{\mathrm{f}}\rangle$ of equation (4). In these interference terms, the factor $\mathcal{C} = \sin 2\alpha$ appears, which is the amount of quantum coherence owned by the pure state $|\psi_{\mathrm{f}}\rangle$ in the basis $\{|\text{wave}\rangle, |\text{particle}\rangle\}$. This is theoretically obtained by using the bona-fide quantifier according to the l_1-norm of coherence for a two-state system [1], defined as $\mathcal{C} = \sum_{i,j\ (i\neq j)} |\rho_{ij}|$, where ρ_{ij} (in our case equal to $\sin\alpha\cos\alpha$) are the off-diagonal terms of the system density matrix.

The coherence of the wave-particle superposition can be directly measured without resorting to the knowledge of the generated state. We can describe the 4-dimensional space spanned by the basis of the initial photon paths $\{|1\rangle, |2\rangle, |3\rangle, |4\rangle\}$ of the preparation part of the setup as

$$|1\rangle = \begin{pmatrix}1\\0\\0\\0\end{pmatrix},\ |2\rangle = \begin{pmatrix}0\\1\\0\\0\end{pmatrix},\ |3\rangle = \begin{pmatrix}0\\0\\1\\0\end{pmatrix},\ |4\rangle = \begin{pmatrix}0\\0\\0\\1\end{pmatrix}. \tag{8}$$

The action of the beam-splitters BS_4 and BS_5 on this basis states is represented by the matrix

$$\mathrm{BS}_{4,5} = \frac{1}{2}\begin{pmatrix}1 & 1 & 0 & 0\\ 1 & -1 & 0 & 0\\ 0 & 0 & 1 & 1\\ 0 & 0 & 1 & -1\end{pmatrix}, \tag{9}$$

which rotates the initial states $\{|1\rangle, |2\rangle, |3\rangle, |4\rangle\}$ into the states, respectively, $\left\{\frac{|1\rangle+|2\rangle}{\sqrt{2}}, \frac{|1\rangle-|2\rangle}{\sqrt{2}}, \frac{|3\rangle+|4\rangle}{\sqrt{2}}, \frac{|3\rangle-|4\rangle}{\sqrt{2}}\right\}$. These rotated states are the eigenstates, with corresponding eigenvalues $\{1, -1, 1, -1\}$, of an operator defined as:

$$\sigma_x^{1234} = \begin{pmatrix}0 & 1 & 0 & 0\\ 1 & 0 & 0 & 0\\ 0 & 0 & 0 & 1\\ 0 & 0 & 1 & 0\end{pmatrix}. \tag{10}$$

This operator corresponds to the simultaneous application of the 2-dimensional first Pauli matrix σ_x, on the pair of states $\{|1\rangle, |2\rangle\}$ and on the pair $\{|3\rangle, |4\rangle\}$. Considering now the operational states of equation (5) and setting $\phi_2 = 0$ (this phase being irrelevant for the particle behaviour), one easily finds

$$\langle\text{wave}|\sigma_x^{1234}|\text{wave}\rangle = \langle\text{particle}|\sigma_x^{1234}|\text{particle}\rangle = 0, \quad \langle\pm|\sigma_x^{1234}|\pm\rangle = \pm 1/\sqrt{2}, \tag{11}$$

where $|\pm\rangle = (|\text{wave}\rangle \pm |\text{particle}\rangle)/\sqrt{2}$: therefore, measuring $\langle\sigma_x^{1234}\rangle$ corresponds to measuring the state along the rotated wave-particle basis $\{|+\rangle, |-\rangle\}$. Moreover, $\langle\sigma_x^{1234}\rangle = \mathrm{Tr}(\sigma_x^{1234}\rho_{\mathrm{f}}) = 0$ for any incoherent (classical) wave-particle state $\rho_{\mathrm{f}} = \cos^2\alpha\,|\text{wave}\rangle\langle\text{wave}| + \sin^2\alpha\,|\text{particle}\rangle\langle\text{particle}|$ and, on the other hand, $\langle\sigma_x^{1234}\rangle = \mathrm{Tr}(\sigma_x^{1234}|\psi_{\mathrm{f}}\rangle\langle\psi_{\mathrm{f}}|) = \sin 2\alpha/\sqrt{2} = \mathcal{C}/\sqrt{2}$ for an arbitrary state of the form $|\psi_{\mathrm{f}}\rangle$ of equation (4). The introduction of the additional beam-splitters BS_4 and BS_5 thus allows the direct measure of the experimental expectation value $\langle\sigma_x^{1234}\rangle$ at the output detectors in terms of the photon detection probabilities, according to the combination $\langle\sigma_x^{1234}\rangle = P_1 - P_2 + P_3 - P_4$. In Fig. 4c of the main text, we report the experimental observations of $\sqrt{2}\langle\sigma_x^{1234}\rangle$ which confirm the generation of a wave-particle quantum superposition with α-dependent coherence, contrasted with the zero mean value (independent of α) measured for an incoherent state ρ_{f} (see Fig. 4d).

It is immediate to theoretically see that, starting from a mixed polarization photon state of the kind $\rho_0 = \cos^2\alpha \left|\mathrm{V}\right\rangle\left\langle\mathrm{V}\right| + \sin^2\alpha \left|\mathrm{H}\right\rangle\left\langle\mathrm{H}\right|$, a mixed incoherent wave-particle state for the photon is finally obtained by the scheme above, namely $\rho_\mathrm{f} = \cos^2\alpha \left|\mathrm{wave}\right\rangle\left\langle\mathrm{wave}\right| + \sin^2\alpha \left|\mathrm{particle}\right\rangle\left\langle\mathrm{particle}\right|$. In the experiment, such a state is effectively achieved by adding a relative time delay in the interferometer paths larger than the photon coherence time to lose quantum interference. Comparisons of the wave-particle morphing and of the coherence witness between the case of coherent wave-particle superposition and the case of mixed wave-particle state are reported in the manuscript (see Fig. 4).

SUPPLEMENTARY NOTE 3: TWO-PHOTON WAVE-PARTICLE ENTANGLED STATE

We now describe the steps leading to the generation of the wave-particle entangled state of two separated photons. The scheme is a parallel doubling of the single-photon scheme of Fig. 1 (see Fig. 2 of the manuscript). In order to give the most general theoretical description of the procedure, let us consider the injection of an initial polarization entangled state of the form

$$\left|\Psi\right\rangle_{\mathrm{AB}} = \cos\alpha \left|\mathrm{VV}\right\rangle + \sin\alpha \left|HH\right\rangle . \tag{12}$$

Then, the photon A (B) is sent to wave-particle toolbox A, top, (B, bottom) as displayed in Fig. 2b of the manuscript. The photons therefore independently follow the same steps described in the section above. For simplicity, we indicate the parameters, optical devices and paths of the bottom wave-particle toolbox (B) with the symbol ($'$).

As a consequence, after the PBS + HWP and PBS$'$ + HWP$'$, the two-photon state becomes

$$\left|\Psi_1\right\rangle = \cos\alpha \left|11'\right\rangle + \sin\alpha \left|22'\right\rangle , \tag{13}$$

where we indicate $|nn'\rangle \equiv |n\rangle|n'\rangle = |n\rangle \otimes |n'\rangle$.

Then, paths 1, $1'$ goes towards BS_1, $\mathrm{BS}_{1'}$, while paths 2, $2'$ bifurcate at BS_2, $\mathrm{BS}_{2'}$, transforming $\left|\Psi_1\right\rangle$ into

$$\left|\Psi_2\right\rangle = \cos\alpha \left[\frac{1}{\sqrt{2}}(\left|1\right\rangle + e^{i\phi_1}\left|3\right\rangle)\right]\left[\frac{1}{\sqrt{2}}(\left|1'\right\rangle + e^{i\phi_{1'}}\left|3'\right\rangle)\right] + \sin\alpha \left[\frac{1}{\sqrt{2}}(\left|2\right\rangle + e^{i\phi_2}\left|4\right\rangle)\right]\left[\frac{1}{\sqrt{2}}(\left|2'\right\rangle + e^{i\phi_{2'}}\left|4'\right\rangle)\right], \tag{14}$$

where ϕ_1, $\phi_{1'}$, ϕ_2 and $\phi_{2'}$ are the relative phases introduced by the phase shifters placed in path 3, $3'$, 4 and $4'$. Successively, paths 1 and 3 are recombined by BS_3 and paths $1'$ and $3'$ are recombined by $\mathrm{BS}_{3'}$, after which the state $\left|\Psi_2\right\rangle$ becomes

$$\left|\Phi\right\rangle_{\mathrm{AB}} = \cos\alpha \left|\mathrm{wave}\right\rangle\left|\mathrm{wave}'\right\rangle + \sin\alpha \left|\mathrm{particle}\right\rangle\left|\mathrm{particle}'\right\rangle , \tag{15}$$

where the states $\left|\mathrm{wave}\right\rangle$ and $\left|\mathrm{particle}\right\rangle$ are defined in equation (5), while the states $\left|\mathrm{wave}'\right\rangle$ and $\left|\mathrm{particle}'\right\rangle$ are defined in the same way, namely

$$\left|\mathrm{wave}'\right\rangle = e^{i\phi_1'/2}\left(\cos\frac{\phi_1'}{2}\left|1'\right\rangle - i\sin\frac{\phi_1}{2}\left|3'\right\rangle\right), \quad \left|\mathrm{particle}'\right\rangle = \frac{1}{\sqrt{2}}\left(\left|2'\right\rangle + e^{i\phi_2'}\left|4'\right\rangle\right). \tag{16}$$

At this stage, that is without the additional beam splitters BS_4, BS_5 and $\mathrm{BS}_{4'}$, $\mathrm{BS}_{5'}$ in each wave-particle toolbox, the entangled state can be measured by photon coincidences $P_{nn'}$ detecting their wave or particle behaviours. In the manuscript, we have performed such a measurement for the case when the two-photon state is maximally entangled (that is, $\alpha = \pi/4$ in equation (15)). The theoretical probabilities corresponding to wave, particle and crossed wave-particle behaviours are as follows. Wave probabilities:

$$\begin{aligned} P_{11'} &= \frac{1}{2}\cos^2\frac{\phi_1}{2}\cos^2\frac{\phi_1'}{2}, \quad P_{33'} = \frac{1}{2}\sin^2\frac{\phi_1}{2}\sin^2\frac{\phi_1'}{2}, \\ P_{13'} &= \frac{1}{2}\cos^2\frac{\phi_1}{2}\sin^2\frac{\phi_1'}{2}, \quad P_{31'} = \frac{1}{2}\sin^2\frac{\phi_1}{2}\cos^2\frac{\phi_1'}{2}. \end{aligned} \tag{17}$$

Particle probabilities:

$$P_{22'} = P_{44'} = P_{24'} = P_{42'} = 1/8. \tag{18}$$

Crossed wave-particle probabilities:

$$P_{12'} = P_{14'} = P_{21'} = P_{23'} = P_{32'} = P_{34'} = P_{41'} = P_{43'} = 0. \tag{19}$$

The experimental results for these sixteen probabilities are plotted in Fig. 5a-d of the manuscript.

When the final beam-splitters in each wave-particle toolbox are used to perform measurements in the rotated wave-particle basis, paths 1 ($1'$) and 2 ($2'$) interfere at BS_4 ($BS_{4'}$) while paths 3 ($3'$) and 4 ($4'$) synchronize at BS_5 ($BS_{5'}$). The overall state of the two photons then becomes

$$|\Phi\rangle_{AB}^{\text{det}} = \cos\alpha\,|\text{wave-det}\rangle\,|\text{wave-det}'\rangle + \sin\alpha\,|\text{particle-det}\rangle\,|\text{particle-det}'\rangle\,, \tag{20}$$

where the states $|\text{wave-det}\rangle$ and $|\text{particle-det}\rangle$ are defined in equation (7), while the states $|\text{wave-det}'\rangle$ and $|\text{particle-det}'\rangle$ are defined in the same way, namely

$$\begin{aligned} |\text{wave-det}'\rangle &= \frac{1}{\sqrt{2}}e^{i\phi_1'/2}(\cos\frac{\phi_1'}{2}|1'\rangle + \cos\frac{\phi_1'}{2}|2'\rangle - i\sin\frac{\phi_1'}{2}|3'\rangle - i\sin\frac{\phi_1'}{2}|4'\rangle), \\ |\text{particle-det}'\rangle &= \frac{1}{2}(|1'\rangle - |2'\rangle + e^{i\phi_2'}|3'\rangle - e^{i\phi_2'}|4'\rangle). \end{aligned} \tag{21}$$

For this state, the coincidence probabilities $P_{nn'} = P_{nn'}(\alpha,\phi_1,\phi_1',\phi_2,\phi_2')$ that a pair of detectors D_n and $D_{n'}$ ($n = 1,2,3,4;\ n' = 1',2',3',4'$) fires are now found to be

$$\begin{aligned}
P_{11'} &= P_{22'} = \frac{1}{4}\cos^2\alpha\cos^2\frac{\phi_1}{2}\cos^2\frac{\phi_1'}{2} + \frac{1}{16}\sin^2\alpha + \frac{1}{8}\sin 2\alpha\cos\frac{\phi_1}{2}\cos\frac{\phi_1'}{2}\cos\left(\frac{\phi_1+\phi_1'}{2}\right), \\
P_{12'} &= P_{21'} = \frac{1}{4}\cos^2\alpha\cos^2\frac{\phi_1}{2}\cos^2\frac{\phi_1'}{2} + \frac{1}{16}\sin^2\alpha - \frac{1}{8}\sin 2\alpha\cos\frac{\phi_1}{2}\cos\frac{\phi_1'}{2}\cos\left(\frac{\phi_1+\phi_1'}{2}\right), \\
P_{13'} &= P_{24'} = \frac{1}{4}\cos^2\alpha\cos^2\frac{\phi_1}{2}\sin^2\frac{\phi_1'}{2} + \frac{1}{16}\sin^2\alpha - \frac{1}{8}\sin 2\alpha\cos\frac{\phi_1}{2}\sin\frac{\phi_1'}{2}\sin\left(\phi_2' - \frac{\phi_1+\phi_1'}{2}\right), \\
P_{14'} &= P_{23'} = \frac{1}{4}\cos^2\alpha\cos^2\frac{\phi_1}{2}\sin^2\frac{\phi_1'}{2} + \frac{1}{16}\sin^2\alpha + \frac{1}{8}\sin 2\alpha\cos\frac{\phi_1}{2}\sin\frac{\phi_1'}{2}\sin\left(\phi_2' - \frac{\phi_1+\phi_1'}{2}\right), \\
P_{31'} &= P_{42'} = \frac{1}{4}\cos^2\alpha\sin^2\frac{\phi_1}{2}\cos^2\frac{\phi_1'}{2} + \frac{1}{16}\sin^2\alpha - \frac{1}{8}\sin 2\alpha\sin\frac{\phi_1}{2}\cos\frac{\phi_1'}{2}\sin\left(\phi_2 - \frac{\phi_1+\phi_1'}{2}\right), \\
P_{32'} &= P_{41'} = \frac{1}{4}\cos^2\alpha\sin^2\frac{\phi_1}{2}\cos^2\frac{\phi_1'}{2} + \frac{1}{16}\sin^2\alpha + \frac{1}{8}\sin 2\alpha\sin\frac{\phi_1}{2}\cos\frac{\phi_1'}{2}\sin\left(\phi_2 - \frac{\phi_1+\phi_1'}{2}\right), \\
P_{33'} &= P_{44'} = \frac{1}{4}\cos^2\alpha\sin^2\frac{\phi_1}{2}\sin^2\frac{\phi_1'}{2} + \frac{1}{16}\sin^2\alpha - \frac{1}{8}\sin 2\alpha\sin\frac{\phi_1}{2}\sin\frac{\phi_1'}{2}\cos\left(\phi_2 + \phi_2' - \frac{\phi_1+\phi_1'}{2}\right), \\
P_{34'} &= P_{43'} = \frac{1}{4}\cos^2\alpha\sin^2\frac{\phi_1}{2}\sin^2\frac{\phi_1'}{2} + \frac{1}{16}\sin^2\alpha + \frac{1}{8}\sin 2\alpha\sin\frac{\phi_1}{2}\sin\frac{\phi_1'}{2}\cos\left(\phi_2 + \phi_2' - \frac{\phi_1+\phi_1'}{2}\right).
\end{aligned} \tag{22}$$

The theoretical predictions of these sixteen probabilities for the generated maximally entangled state of the experiment reported in the manuscript, can be retrieved by fixing $\alpha = \pi/4$. The experimental plots are reported in Fig. 5e-h of the manuscript. We recall that the detection of these probabilities corresponds to measuring each photon of the entangled pair along the coherent superposition of wave and particle behaviours.

We point out that the third terms of all the coincidence probabilities of equation (22) are identically zero (that is, independently of the values of the phases) if and only if there is no quantum entanglement between the wave and particle degrees of freedom of the two photons. In fact, all these terms contain the factor $C = \sin 2\alpha = 2\sin\alpha\cos\alpha$, where C is the standard concurrence quantifying the entanglement of the state $|\Phi\rangle_{AB}$ of equation (15) in the two-photon basis $\{|\text{wave}\rangle|\text{wave}'\rangle, |\text{wave}\rangle|\text{particle}'\rangle, |\text{particle}\rangle|\text{wave}'\rangle, |\text{particle}\rangle|\text{particle}'\rangle\}$ [2]. A first way to experimentally assess the concurrence of $|\Phi\rangle_{AB}$ is by suitably combining the detected coincidence probabilities above, assuming the knowledge of the state. In particular, setting $\phi_2 = \phi_2' = 0$ (being these phases unimportant in the creation of the wave-particle entangled state), one finds $C/4 = (P_{11'} - P_{12'}) + (P_{13'} - P_{14'}) + (P_{31'} - P_{32'}) + (P_{33'} - P_{34'})$. However, to quantitatively assess the theoretical predictions for the generated state, it is sufficient to employ the quantity

$$\mathcal{E} = P_{11'} - P_{12'} = P_{22'} - P_{21'} = \frac{C}{4}\cos\frac{\phi_1}{2}\cos\frac{\phi_1'}{2}\cos\left(\frac{\phi_1+\phi_1'}{2}\right), \tag{23}$$

which is identically zero if and only if the wave-particle two-photon state is separable (unentangled): $\alpha = 0$ ($|\text{wave}\rangle \otimes |\text{wave}'\rangle$), $\alpha = \pi/2$ ($|\text{particle}\rangle \otimes |\text{particle}'\rangle$). This quantity would be identically zero also for a mixture of two-photon wave and particle states like $\rho_{AB} = \cos^2\alpha|\text{wave}\rangle\langle\text{wave}| \otimes |\text{wave}'\rangle\langle\text{wave}'| + \sin^2\alpha|\text{particle}\rangle\langle\text{particle}| \otimes |\text{particle}'\rangle\langle\text{particle}'|$, since the third terms in the probabilities do not appear at all ($C = 0$). For the generated entangled state in the experiment, that is for $|\Phi\rangle_{AB}$ of equation (15) with $\alpha = \pi/4$ and thus $C = 1$, fixing $\phi_1' = 0$, this quantity reduces to $\mathcal{E} = (1/4)\cos^2(\phi_1/2)$. Experimental measurements of the latter are reported in Fig. 5i-j of the manuscript and confirm the theoretical predictions.

We can also provide a further stronger test of the creation of the wave-particle entanglement $|\Phi\rangle_{\mathrm{AB}}$ of equation (15) which does not resort to the knowledge of the state itself. This test is realized by directly measuring the expectation value of the entanglement witness defined, in the two-photon path basis $\{\{|1\rangle, |2\rangle, |3\rangle, |4\rangle\} \otimes \{|1'\rangle, |2'\rangle, |3'\rangle, |4'\rangle\}\}$, as

$$\mathcal{W} = \mathbb{1} - 2[\sigma_x^{1234} \otimes (\sigma_x^{1234})'] - [\sigma_z^{1234} \otimes (\sigma_z^{1234})'], \tag{24}$$

where $\mathbb{1} = \mathbb{1} \otimes \mathbb{1}'$ is the identity matrix and

$$\sigma_z^{1234} = \begin{pmatrix} 1 & 0 & 0 & 0 \\ 0 & -1 & 0 & 0 \\ 0 & 0 & 1 & 0 \\ 0 & 0 & 0 & -1 \end{pmatrix}. \tag{25}$$

The measurement basis of σ_x^{1234}, as already explained in the Supplementary Note 2 above, is the rotated one after the insertion of beam-splitters BS_4 and BS_5. The measurement basis of σ_z^{1234} is instead simply that of the initial paths $\{|1\rangle, |2\rangle, |3\rangle, |4\rangle\}$ exiting the preparation part of the single-photon toolbox and directly going to the detectors. It is immediate to see that $\langle \mathrm{wave}|\sigma_z^{1234}|\mathrm{wave}\rangle = 1$ and $\langle \mathrm{particle}|\sigma_z^{1234}|\mathrm{particle}\rangle = -1$, where the wave and particle states are defined in equation (5). In order that $\mathcal{W}$ is a faithful entanglement witness, its expectation value $\langle\mathcal{W}\rangle = \mathrm{Tr}(\mathcal{W}\rho)$ must satisfy the requirement: $\mathrm{Tr}(\mathcal{W}\rho_{\mathrm{s}}) \geq 0$ for any two-particle separable state ρ_{s} of wave-particle states, so that whenever $\mathrm{Tr}(\mathcal{W}\rho_{\mathrm{e}}) < 0$ the state ρ_{e} is entangled in the photons wave-particle behaviour [3]. In the following, we show that $\mathcal{W}$ indeed fulfills this property.

A general bipartite separable state ρ_{s} is given by a convex sum of product states of the kind [2,3]: $\rho_{\mathrm{s}} = \sum_i p_i \rho_i \otimes \rho_i'$, where $\sum_i p_i = 1$, while $\rho_i = \sum_k a_k |\psi_k^i\rangle\langle\psi_k^i|$ and $\rho_i' = \sum_{k'} b_{k'} |\varphi_{k'}^i\rangle\langle\varphi_{k'}^i|$ are generic single-particle states with $\sum_k a_k = \sum_{k'} b_{k'} = 1$. Describing the single-particle space as spanned by the two operational states $|\mathrm{wave}\rangle \equiv |\mathrm{w}\rangle$ and $|\mathrm{particle}\rangle \equiv |\mathrm{p}\rangle$, an arbitrary single-particle pure state can be expressed as

$$|\psi\rangle = \cos\theta|\mathrm{w}\rangle + e^{i\gamma}\sin\theta|\mathrm{p}\rangle \quad \Rightarrow \quad |\psi\rangle\langle\psi| = \begin{pmatrix} \cos^2\theta & e^{i\gamma}\sin\theta\cos\theta \\ e^{-i\gamma}\sin\theta\cos\theta & \sin^2\theta \end{pmatrix}. \tag{26}$$

Since it is trivial that $\langle\mathbb{1}\rangle = 1$ whatever the two-particle state, let us focus on the second and third terms of equation (24). We first notice that

$$\mathrm{Tr}[\sqrt{2}\sigma_x^{1234}|\psi\rangle\langle\psi|] = \sqrt{2}\langle\psi|\sigma_x^{1234}|\psi\rangle = \cos\gamma\sin 2\theta, \quad \mathrm{Tr}[\sigma_z^{1234}|\psi\rangle\langle\psi|] = \langle\psi|\sigma_z^{1234}|\psi\rangle = \cos 2\theta. \tag{27}$$

By exploiting the well-known properties of the Kronecker (tensor) product of matrices $(A \otimes B)(C \otimes D) = (AC) \otimes (BD)$, $\mathrm{Tr}(A \otimes B) = \mathrm{Tr}(A)\mathrm{Tr}(B)$ and the linearity of trace, for any couple of pure states $|\psi\rangle$, $|\psi'\rangle$ of the two particles we obtain that

$$\begin{aligned} &\mathrm{Tr}[(2\sigma_x^{1234} \otimes (\sigma_x^{1234})' + \sigma_z^{1234} \otimes (\sigma_z^{1234})')(|\psi\rangle\langle\psi| \otimes |\psi'\rangle\langle\psi'|)] = \\ &= 2\mathrm{Tr}[\sigma_x^{1234}|\psi\rangle\langle\psi|]\mathrm{Tr}[(\sigma_x^{1234})'|\psi'\rangle\langle\psi'|] + \mathrm{Tr}[\sigma_z^{1234}|\psi\rangle\langle\psi|]\mathrm{Tr}[(\sigma_z^{1234})'|\psi'\rangle\langle\psi'|] = \\ &= (\cos\gamma\sin 2\theta)(\cos\gamma'\sin 2\theta') + \cos 2\theta\cos 2\theta' \leq \sin 2\theta\sin 2\theta' + \cos 2\theta\cos 2\theta' = \cos[2(\theta - \theta')] \leq 1. \end{aligned} \tag{28}$$

This first inequality is the basic one for our demonstration. In fact, considering now the general separable state ρ_{s} as defined above, we have

$$\begin{aligned} &\mathrm{Tr}[(2\sigma_x^{1234} \otimes (\sigma_x^{1234})' + \sigma_z^{1234} \otimes (\sigma_z^{1234})')\rho_{\mathrm{s}}] = \sum_i p_i \sum_k a_k \sum_{k'} b_{k'} \mathrm{Tr}[(2\sigma_x^{1234} \otimes (\sigma_x^{1234})' + \\ &+ \sigma_z^{1234} \otimes (\sigma_z^{1234})')(|\psi_k^i\rangle\langle\psi_k^i| \otimes |\varphi_{k'}^i\rangle\langle\varphi_{k'}^i|)] \leq \sum_i p_i \sum_k a_k \sum_{k'} b_{k'} = 1, \end{aligned} \tag{29}$$

where in the last line we have just used the result of equation (28) and the normalization of all the coefficients. As a consequence of equation (29), we finally get for the operator of equation (24)

$$\mathrm{Tr}(\mathcal{W}\rho_{\mathrm{s}}) = 1 - \mathrm{Tr}[(2\sigma_x^{1234} \otimes (\sigma_x^{1234})' + \sigma_z^{1234} \otimes (\sigma_z^{1234})')\rho_{\mathrm{s}}] \geq 0, \tag{30}$$

for any separable state ρ_{s} of wave-particle states. The theoretical prediction of its mean value for the wave-particle entangled state $|\Phi\rangle_{\mathrm{AB}}$ of equation (15) is $\mathrm{Tr}[\mathcal{W}|\Phi\rangle_{\mathrm{AB}}\langle\Phi|] = -1$. Therefore, $\mathcal{W}$ is a faithful entanglement witness.

Being the diagonal forms of the two local observables σ_x^{1234}, σ_z^{1234} equal (see equations (10) and (25)) with different measurement basis (eigenstates), it is immediate to realise that the experimental measure of both $\langle\sigma_x^{1234} \otimes (\sigma_x^{1234})'\rangle$ and $\langle\sigma_z^{1234} \otimes (\sigma_z^{1234})'\rangle$ is provided by the same combination of the detected coincidence probabilities as follows: $P_{11'} - P_{12'} + P_{13'} - P_{14'} - P_{21'} + P_{22'} - P_{23'} + P_{24'} + P_{31'} - P_{32'} + P_{33'} - P_{34'} - P_{41'} + P_{42'} - P_{43'} + P_{44'}$. In the main text we report the expectation values of $\mathcal{W}$ measured in the experiment for the different phases, namely: $\langle\mathcal{W}\rangle = -0.699 \pm 0.041$ ($\phi_1 = \phi_1' = 0$); $\langle\mathcal{W}\rangle = -0.846 \pm 0.045$ ($\phi_1 = \phi_1' = \pi$); $\langle\mathcal{W}\rangle = -0.851 \pm 0.041$ ($\phi_1 = \pi$, $\phi_1' = 0$); $\langle\mathcal{W}\rangle = -0.731 \pm 0.042$ ($\phi_1 = 0$, $\phi_1' = \pi$). These observations ultimately confirm the effective generation of a wave-particle entanglement.

We notice that by changing the initial polarization entangled state, different wave-particle entangled states can be created. For example, $\frac{1}{\sqrt{2}}(|\mathrm{VH}\rangle + |\mathrm{HV}\rangle)$ leads to $\frac{1}{\sqrt{2}}(|\mathrm{wave}\rangle\,|\mathrm{particle}'\rangle + |\mathrm{particle}\rangle\,|\mathrm{wave}'\rangle)$.

SUPPLEMENTARY REFERENCES

[1] Streltsov, A., Adesso, G. & Plenio, M. B. Quantum coherence as a resource. Preprint at http://arxiv.org/abs/1609.02439 (2016).

[2] Horodecki, R. Horodecki, P., Horodecki, M. & Horodecki, K. Quantum entanglement. *Rev. Mod. Phys.* **81**, 865–942 (2009).

[3] Gühne, O. & Tóth, G. Entanglement detection. *Phys. Rep.* **474**, 1–75 (2009).
